
JOURNAL INFORMATION
==============================
NLM Title Abbreviation: ScientificWorldJournal
Journal ID: ScientificWorldJournal
NLM Journal ID: 101131163
Journal ID: TSWJ

The Scientific World Journal

ISSN: 2356-6140
EISSN: 1537-744X
Publisher: Wiley

ARTICLE INFORMATION
==============================
PMCID: PMC6084238
DOI: 10.1100/tsw.2001.426
Article ID: 818963
Article version: 1
Subjects: Extended Abstract

Peripheral Nerve Injury and CGRP Receptor Antagonist Modulate RCP-1 Expression in Primary Sensory Neurons and in Neurons of the Spinal Cord

Ma W. 1
Chabot J.-G. 1
Powell K. J. 2
Jhamandas K. 2
Dickerson I. 3
Quirion R. 1
1 Douglas Hospital Research Center and Department of Psychiatry, McGill University, Verdun, QC, Canada H4H 1R3,
2 Department of Pharmacology and Toxicology, Faculty of Health Science, Queens University, Kingston, ON, Canada K7L 3N6,
3 Department of Physiology and Biophysics, University of Miami, FL, USA
Publication date: 2001
Electronic publication date: 2001 Nov 9
Volume: 1
First page: 43
Last page: 43
Copyright: Copyright © 2001 W. Ma et al.
Copyright year: 2001
License: This is an open access article distributed under the Creative Commons Attribution License, which permits unrestricted use, distribution, and reproduction in any medium, provided the original work is properly cited.
License URL: https://creativecommons.org/licenses/by/3.0/

Funding: Canadian Institutes of Health Research

==============================
4th International Meeting on Calcitonin Gene-Related Peptide (CGRP)
*TheScientificWorld* (2001) 1(S1), 43
ISSN 1532-2246; DOI 10.1100/tsw.2001.426

### PERIPHERAL NERVE INJURY AND CGRP RECEPTOR ANTAGONIST MODULATE RCP-1 EXPRESSION IN PRIMARY SENSORY NEURONS AND IN NEURONS OF THE SPINAL CORD

W. Ma[1], J.-G. Chabot[1], K.J. Powell[2], K. Jhamandas[2], I. Dickerson[3], and R. Quirion[1]
[1]Douglas Hospital Research Center and Department of Psychiatry, McGill University, Verdun, QC, Canada, H4H 1R3; [2]Department of Pharmacology and Toxicology, Faculty of Health Science, Queens University, Kingston, ON, Canada K7L 3N6; [3]Department of Physiology and Biophysics, University of Miami, FL

Calcitonin gene-related peptide (CGRP) receptors appear to be rather unique among G-protein receptor superfamily since they may require accessory proteins to be functional. For example, a CGRP receptor clone behaves as a CGRP1 receptor subtype in presence of the RAMP-1 accessory protein but as an adrenomedullin receptor when coexpressed with RAMP-2. Another putative protein bearing no sequence homology to the RAMPs has been cloned and named CGRP receptor component or RCP. This protein is involved in the signal transduction pathway of the CGRP receptors. Previously, using highly specific RCP-1 antibody, we immunocytochemically demonstrated the localization of RCP-1 immunoreactivity in the brain and peripheral nervous system of the rat. The aim of this study was to determine if RCP-1 expression in primary sensory neurons and spinal motoneurons could be modulated by partial sciatic nerve injury, a model for neuropathic pain, or by intrathecal administration of $CGRP_{8-37}$, a CGRP receptor antagonist. We found that partial sciatic nerve injury reduced RCP-1 expression in neurons of both dorsal and ventral horns of the lumbar spinal cord. Interestingly, following intrathecal daily injection of $CGRP_{8-37}$ (4 µg/day for 7 days), RCP-1 immunoreactive axons and neurons were significantly increased in both dorsal and ventral horns of the rat lumbar spinal cord. These data suggest that RCP-1 expression in the primary sensory neurons and spinal motoneurons is subject to modulation by peripheral nerve injury as well as CGRP receptor activation. It could be used as a reliable marker to represent CGRP receptor. Supported by grant from CIHR.
